# Cholesterol-lowering effects and safety assessment of *Lactobacillus* spp. in vivo and in vitro testing for human use as probiotic from the dairy product in Egypt

**DOI:** 10.1186/s43141-022-00423-3

**Published:** 2022-10-18

**Authors:** Mostafa G. Fadl, Zenat Kamel

**Affiliations:** 1 Nuclear Materials Authority, P.O. Box 530, El Maadi, Cairo, Egypt; 2 Faculty of Science, Microbiology & Botany Department, Cairo University, Cairo, Egypt

**Keywords:** Safety assessment, *Lactobacillus*, Enzymatic activity, Probiotic, Bile salt Hydrolysis gene

## Abstract

**Background:**

The toxicity profile of lactobacilli may be strain dependent, so it should be considered for safe utilization of probiotics. Further, in vivo studies are necessary to evaluate their safety.

**Result:**

The ability of various probiotic strains to hydrolyze bile salts has been confirmed without noticeable hemolytic activity. Results revealed the presence of α-glucosidase, β-glucosidase, α-galactosidase, and β-galactosidase activity in all investigated isolates, while none of the isolates produced the carcinogenic enzyme β-glucuronidase. The probiotic strains exhibited remarkable cholesterol-lowering impact. Also, we found no evidence of chronic toxicity under the experimental conditions based on gross pathological examination of the viscera and study of the spleen and liver weight ratios. These findings indicated that the investigated strains, either alone or combined with their metabolites, had no obvious adverse effect on the mice's general health status.

**Conclusion:**

There is prove that the investigated probiotic strains are safe to be utilized for enhancing of the growth performance and are free of adverse side effects.

## Background

### Safety aspects of probiotics of LAB

The acidification of milk depends on efficient conversion of the milk-sugar lactose to lactic acid which contributes to the extended shelf-life fermented milk products by preventing the outgrowth of pathogenic and spoilage organisms. The lactic acid bacteria (LAB) are belonging to probiotics that have several beneficial effects on human health [1]. It has been suggested that supplementation of dairy products with *Lactobacillus* spp. exerts a significant influence on microbial metabolism in the colon by reducing fecal β-glucuronidase and nitroreductase activities related to the release and formation of toxic compounds in the colon. The safety of probiotic items is evaluated based on the phenotypic and genotypic characteristics and microbial measures [2, 3]. The majority of data are from opportunistic pathogenic enterococci, while few reports on lactococci and lactobacilli. Vancomycin-resistant enterococci (VRE) are disseminated via the food chain, which have emerged in the last decade as a frequent cause of nosocomial infections [4, 5]. A previous study investigated the in vitro susceptibility of *Enterococcus faecium* strains isolated from food products to a diverse array of antibiotics [6]. The phenotypic analysis was employed to determine their resistance to a diverse array of antibiotics; enterococci are commonly considered intrinsically resistant to low levels of gentamicin, enterococci isolated from milk and cheese were screened for gentamicin resistance [7]. Numerous dairy isolates have been shown to have a high level of gentamicin resistance. The molecular components of gentamicin resistance in *Enterococcus* isolates from creatures, foods, and patients were determined [8]. It has been proposed that enterococci isolated from humans, retail food, and cultivated creatures have similar gentamicin resistance. Moreover, the spread of gentamicin-resistant enterococci from creatures to people through the food supply was demonstrated. Tetracycline resistance could be linked to the presence of tet (M) genes in *enterococcal* isolates [9].

Given the increasing promotion of probiotic strains of *Lactobacillus* and *Bifidobacterium* in consumer products, we anticipate that widespread probiotic utilization may enhance corpulence by altering the intestinal microbiome [10, 11]. On the other hand, for at least 30 years, probiotics have been employed to manipulate the gut microbiota to boost growth in farm animals. Indeed, *Lactobacillus acidophilus* is often commonly used in agriculture. All this information unequivocally proposes that *Lactobacillus*-containing probiotics (LCP) may affect weight control in people and creatures. Numerous studies have examined the effects of *Lactobacillus*-containing probiotics (LCP) on weight, but subsequent information indicates that this effect is, at best, species-specific [11–13].

## Methods

### Probiotic characterization by molecular tools

The bile salt hydrolase gene was detected in superior isolates by polymerase chain reaction (PCR) using bsh primers (5′ GGATTGTGTATTGCGGGATT 3′) and (5′ AGTCCGCCCATTCCTCTACT 3′) following the method described by [14]. The PCR reaction cycles were as follows: initial denaturation at 95 °C for 4 min followed by 35 consecutive cycles of 94 °C for 1 min, 55 °C for 40 s, 72 °C for 2 min and final extension of 72 °C for 10 min. The resultant PCR products were analyzed by agarose gel electrophoresis.

### Studying enzymatic activity of *Lactobacillus* isolates

The API ZYM pack (bio-Mérieux, France) was used to investigate the enzymatic profiles of *Lactobacillus* isolates according to the manufacturer instructions. Each isolate was grown overnight in MRS broth at 37 °C. After incubation, cells were collected by centrifugation and resuspended to be inoculated into API ZYM kit microcupules. Afterwards, the inoculated strips were covered and incubated at 37 °C for 4 °h. Subsequently, 30 °μl of each reagent (ZYM A and ZYM B; BioMerieux, France) were added to each microcupules and incubated for 5 min. Results were recorded according to the manufacturer’s instructions using the scale from 0 to 5 based on the visual color intensity.

### Antibiotics susceptibility of *Lactobacillus* isolates

The Kirby-Bauer disk diffusion method was used to screen lactobacilli isolates for antimicrobial susceptibility to 15 antibiotics: ampicillin/sulbactam, amoxicillin/clavulanic acid, clarithromycin, erythromycin, nalidixic acid, trimethoprim/sulphamethoxazole, ciprofloxacin, tetracycline, vancomycin, and rifampicin. According to clinical laboratory standards institute. In brief, a few fresh colonies of each strain were picked with a wire loop and incubated in MRS broth. The inoculated tubes were then incubated at 35 °C for 2 to 5 h till the turbidity equivalent to 0.5 McFarland standards was developed. The suspension is then diluted (1:10) with saline to yield a uniform bacterial suspension. Muller Hinton agar plates were then inoculated with bacterial suspension using a sterile cotton swab. The discs were firmly applied to the surface of the agar plate using aseptic techniques with centers at least 24 mm apart. The plates were incubated at 35 °C and examined after 16–18 h.

### In vivo studies of *Lactobacillus* isolates

In this investigation, three probiotic *Lactobacillus* spp. isolates identified as *L. case*, *L. lactis*, and *L. acidophilus* were employed in animal feed. Cultures grown in MRS broth were concentrated by centrifugation, and the cell pellets were resuspended in a diluent after three washes to give a final concentration of 10^8^ CFU/ml. These preparations were then added to the drinking water given to the mice at a final concentration of 20% (v/v). In another set (control), drinking water without bacterial suspensions given to the mice under the same conditions.

### Animals and diets

Mice weighing 13–17 g from a private laboratory (creative lab) were housed in groups of 5 males and 5 females per cage. A regular light-dark cycle and a controlled atmosphere with a temperature of 22 °C and relative humidity of 55% were maintained throughout the study. The animals were given free access to feed, which may be either a barley-based basal diet or an enriched conventional feed (mice were provided with drinking water).

### Experimental design

In this study, five groups of mice were evaluated to assess the safety of probiotic isolates. Namely, G1 was subjected to a commercial strain of *L. plantarum* (positive control); G2 received drinking water without any probiotics (negative control); G3 was subjected to *L. Acidophillus*; G4 was subjected to *L. casei* and G5 was subjected to *L. lactis*. The mice were acclimatized to the experimental conditions for 24 h. Supplemented drinking water and feed were changed daily. The treatments for the toxicology study lasted for 4 weeks, whereas the growth-promoting treatment lasted 10 days. Hair luster was observed at the end of the treatment period, and each mouse’s body weight was recorded daily. All animals were murdered via cervical separation on the final day of the test.

### Evaluation of growth performance

Daily body weight measurements were taken using a mouse balance (Sartorius). The weight gain (WG) was expressed as the mean of each mouse's final weight minus its initial weight. The specific growth rate (SGR) was expressed as the daily weight gain. Feed intake (FI) and water intake were monitored daily for each cage and expressed per animal for the total period by dividing feed or water consumption by the number of animals. The consumption index (CI) was calculated as the ratio of FI/WG [15]. Hemoglobin and liver enzyme levels were measured to determine side effects.

## Results and discussions

### Probiotic characterization by molecular tools

**Fig. 1:**
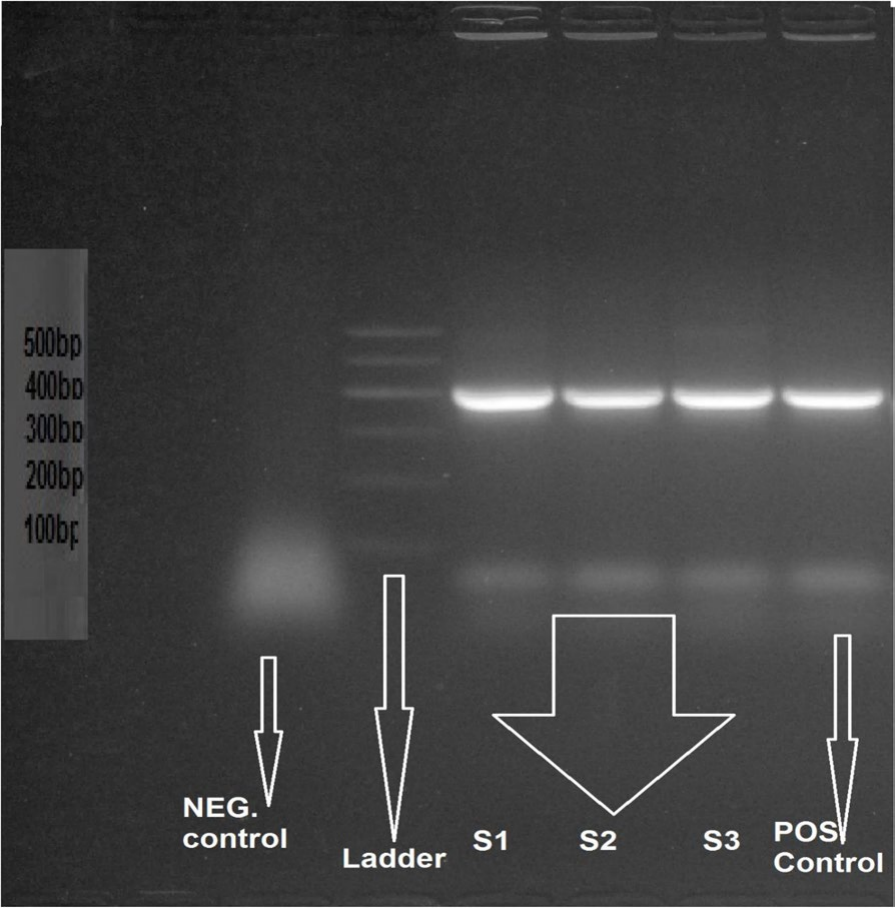
Gel electrophoresis for Bile salt hydrolysis gene S1, S2, and S3 bands are probiotic bacterial isolates, beside band of positive control

A bile salt hydrolase gene from *L. Plantarum* was utilized as a potential food-grade determination marker to develop a novel vector for lactic acid microbes [16]. Using a primer for the bile salt hydrolysis gene to confirm superior isolates’ probiotic characteristics resulted in a positive result (Fig. 1), demonstrating that isolates can hydrolyze bile salt as a probiotic trait [14].

### The cholesterol-lowering effects of lactic acid bacteria and mechanism

The ability to hydrolyze bile salts has been added to the criteria for determining probiotic strains with cholesterol-lowering effects, as numerous non-deconjugating strains were unable to evacuate cholesterol from the culture medium [17]. Several investigations on the hypocholesterolemic effects of BSH-producing lactic acid bacteria in vivo have prompted increased interest in maintaining cholesterol levels in healthy individuals or conceivable applications for hypercholesterolemic individuals. In this case, BSH-positive bacterial cells shows potential for controlling blood cholesterol levels. In any case, additional considerations revealed that probiotics had negligible effects on cholesterol-lowering impacts, hence casting doubt on the hypocholesterolemic claim. Many researchers proposed that probiotics have cholesterol reduction effects. However, the mechanism of this effect could not be explained definitely. Two hypotheses are trying to explain the mechanism. One of them is that bacteria may bind or incorporate cholesterol directly into the cell membrane which confirmed by evidences saying that (LAB) lactic acid bacteria are non-pathogenic and safe microbes that generate numerous mature food products. It transforms glucose into lactic acid, ethanol, and CO_2_, all of which improve the quality, surface, and smell of fermented items [18]. Microbial cells normally absorb metal particles due to their utility for creating cell layers [19]. LAB has recently been applied during metal particle restriction, despite the results of reviews investigating the coupling capacity of metal particles of different microorganisms. Have performed a broad review of past studies on the absorption limits of individual clusters of microorganisms [20]. The other one is, bile salt hydrolysis enzymes deconjugate the bile salts, which are more likely to be exerted, resulting in increased cholesterol breakdown [2, 21, 22]. Using a primer for the bile salt hydrolysis gene to confirm superior isolates’ probiotic characteristics resulted in a positive result, demonstrating that isolates can hydrolyze bile salt as a probiotic trait [14] confirmed by a study on the reduction of cholesterol showed that *Lactobacillus reuteri* decreased total cholesterol by 38% when given to mice for 7 days at the rate of 10^4^ cells/day. This dose of *Lactobacillus reuteri* caused a 40% reduction in triglycerides and a 20% increase in the ratio of high-density lipoprotein to low-density lipoprotein without bacterial translocation of the native microflora into the spleen and liver [23]. Report to provide quantitative evidence of the dose-dependent effect of *Lactobacillus* sp. Agreeing with Shiuh et al., with a minimal effective dose of 6 × 10^8^ CFU for 3 days, without any exception, the fecal rotavirus concentrations of all eight patients in the high-dose group declined by 86% after 3 days when compared with those before administration [17, 24, 25].

### Enzymatic activities of *Lactobacillus* isolates

The clarification of *Lactobacillus* bacterial enzymes may aid in its identification, taxonomic placement, and increased utilization in the dairy industry and improve our understanding of its effect on bacterial metabolism and gut function. In the present study, API ZYM kit was used to detect 19 different hydrolases from *Lactobacillus* spp. Results revealed the presence of α-glucosidase, β-glucosidase, α-galactosidase, and β-galactosidase in all investigated isolates, while none of the isolates produced the carcinogenic protein β-glucuronidase. The enzymatic profile of the investigated isolates is shown in Table 1. These results match those found in a previous study [26, 27]. Similarly, β-galactosidase was found in *Lactobacillus* isolated from fermented oil, as previously reported [26]. This rapid and simple method might be useful for classifying probiotic bacteria [6]. Enzyme generation by isolates was an imperative measure in its determination since microorganisms can deliver carcinogenic enzymes such as β-glucuronidase [28].

**Table 1:** Enzyme activity of isolated *Lactobacillus* spp.

Enzyme	Enzyme activity of isolates								
	SI	S2	S3	S4	S5	S6	S7	B.M	S8
**Control**	0	0	0	0	0	0	0	0	0
**Alkaline phosphatase**	0	0	0	0	0	0	0	0	0
**Esterase**	0	0	0	0	0	0	0	0	0
**Esterase lipase**	0	0	0	0	0	0	0	0	0
**Lipase**	0	0	0	0	0	0	0	0	0
**Leucinearylamidase**	2	2	2	2	2	2	2	3	2
**Valinearylamidase**	0	0	0	0	0	0	0	0	0
**Cystinearylamidase**	1	1	1	1	2	1	1	2	1
**Trypsin**	0	0	0	0	0	0	0	0	0
**α-Chymotrypsin**	0	0	0	0	0	0	0	0	0
**Acid phosphatase**	2	2	2	2	2	2	2	2	2
**Naphthol-AS-BI-phosphohydrolase**	5	5	5	5	5	5	5	5	5
**α-Galactosidase**	2	2	2	2	2	2	2	2	2
**β-Galactosidase**	5	5	5	5	5	5	5	5	5
**β-Glucuronidase**	0	0	0	0	0	0	0	0	0
**α-Glucosidase**	2	2	2	2	2	2	1	2	1
**β-Glucosidase**	2	2	2	2	2	2	2	2	2
**N-Acetyl-β-glucosaminidase**	3	3	3	3	3	3	3	3	3
**α-Mannosidase**	0	0	0	0	0	0	0	0	0
**α-Fucosidase**	0	0	0	0	0	0	0	0	0

*0* no activity, *1* low activity, *2–3* moderate activity, *4–5* high activity

### Antibiotics susceptibility of *Lactobacillus* isolates

Lactic acid bacteria are broadly utilized as probiotics or starter cultures and can be a repository of antimicrobial resistance genes. Thus, using LAB increase the possibility of antibiotic resistance genes being transferred to lactic acid microorganisms and other pathogenic microbes. In recent years, there has been an increased emphasis on nutrition as a vehicle for antimicrobial resistance genes [29–31]. A recent study provided an overview of the techniques available for studying mobile DNA transfer in microbial communities [32]. However, very few systematic studies have been conducted on LAB acquired antibiotic resistance through food.

Exchange of resistance to antimicrobial substances is a basic component in *Lactobacillus* adaptation and survival in particular environments. Among the resistance components in use, protein inactivation of the antimicrobial, restricted antimicrobial effect, dynamic trade of anti-microbials, or target alteration may be highlighted [33]. For a variety of lactobacilli, exceptionally tall frequencies of unconstrained transformations have been observed in response to nitrofurazone, kanamycin, and streptomycin [9]. Another study was performed to establish the levels of susceptibility of *Lactobacillus* spp. to various antimicrobial agents, revealing species dependence [34]. The resistance spectrum of *Bifidobacterium* was previously described [31]. The investigated bifidobacteria (probiotics) were susceptible to many antibiotics. There was discovered resistance, some of it most likely intrinsic [35]. It was reported that *L. lactis* strains were sensitive to amikacin, ampicillin, 1st generation cephalosporin, and many antibiotics [4]. Numerous drug efflux proteins were found in *L. lactis* subsp. *lactis* [34], including an ABC transporter and a proton motive force-dependent drug transporter. The resistance against vancomycin is due to the proximity of d-alanine: d-alanine ligase-related proteins [36]. Fifteen strains of *Streptococcus thermophiles* isolated from yogurt cultures showed varying resistance levels to different antibiotics [37]. Also, *S. thermophile* strains were previously examined to determine their antibiotic resistance patterns and plasmid carriage [3]. Most strains of *S. thermophilus* were resistant to gentamicin. However, no correlation was observed between the resistance to antibiotics and the occurrence of plasmids in some strains. The antibiotic resistance and incidence of *Enterococcus* species were studied in a white cheese [38]. The Scientific Committee on Animal Nutrition (SCAN) issued a 2002 opinion on the criteria to determine the safety of microorganisms resistant to antibiotics of human clinical and veterinary importance (European Commission 2002). It has been reported that all bacterial products intended for use as auxiliary substances must be inspected to determine the component strain(s)’ resistance to a considerable extent of antimicrobial [39]. Such tests must be conducted consistently and according to internationally accepted and standardized procedures [40, 41].

### Effect of supplementation with *Lactobacillus* cultures on body weight

This study focused on mice’s safety, health, and growth performance receiving these *Lactobacillus* spp. daily for 2 or 4 weeks. With a conventional diet, there was no significant difference in the body weight among groups. Regardless of the lactobacilli strains utilized, no significant change in WG was observed while using a barley diet. The difference between the WG in the mice given water supplemented with *lactobacilli* strains and in those receiving waters in previous table groups 1, 3, 4, and 5 supplemented with different species of *Lactobacillus* and group 2 as control no significant difference in body weight and hemoglobin content. There was no significant difference between feeding of pre- and post-*lactobacillus* spp. A previous study demonstrated a significant beneficial effect on weight loss and a 45% lower risk of becoming press inadequate after 12 months of probiotic- and prebiotic-fortified milk consumption while no impact was noticed on person press insufficiency markers, B12 and folate included initially a significant proportion of children who were anemic and B12 and folate-deficient [39, 42]. In a previous study of no anemic healthy young women with low iron status, viable lyophilized *Lactobacillus Plantarum* added to 1 test meal did not enhance iron absorption [43–45].

### Growth performance parameter after feeding with *Lactobacillus*

#### Effect of feeding with probiotic *lactobacillus* spp. on growth and liver enzyme in vivo

Although it has been shown that most *Lactobacillus* species (e.g., *L. acidophilus*, *L. lactis* and *L. casei*, and reference strain *lactobacillus Plantarum*) are non-pathogenic and do not cause acute oral toxicity for animals (see Tables 2, 3, and 4), it has been reported that it is important to check the safety of each probiotic strain, as the toxicity profile may be strain-dependent [17]. For example, it has been demonstrated an increase in the liver or spleen weight ratios of mice fed a strain *of L. Plantarum* (dead or live cells) [42]. Young mice were used in this study to reinforce any potential toxic impact.

**Table 2:** No. of isolate resistance to antibiotics of the representative strains of lactic acid bacteria isolated from dairy products

Antibiotic	***L. lactis***	***L. casei***	***L. acidophilus***	No. of isolates
Ampicillin	1	3	4	8
Augmentin	1	2	3	6
Cefoxitin	1	3	3	7
Cephalotoxin	1	3	4	8
Oxacillin	1	2	2	5
Vancomycin	1	3	4	8
Teicoplanin	1	3	4	8
Cloranphenicol	1	2	3	6
Clindamycin	1	3	3	7
Rifampicin	1	3	2	6
Tetracycline	0	0	0	0
Kanamycin	1	3	4	7
Ciprofloxacin	0	2	3	5
Nitrofurantoin	1	1	2	4
Trimethoprim	1	3	4	7

**Table 3:** The effect of *lactobacillus* cultures on body weight in experimental mice for 10 days

Time (day)	Body weight (g)				
	G1	G2	G3	G4	G5
@ Zero time	14.6	13.25	15.45	15.75	16.85
1	15.25	14.5	16.05	16.2	17.5
2	15.77	16.6	16.6	16.8	18.3
3	15.9	15.15	17	16.06	18.8
4	16.4	17.75	17.25	18.6	19.56
5	15.85	16.4	18.722	19.41	19.81
6	16.75	16.5	18.5	20	19.21
7	18.93	19.5	21.5	23.6	17.125
8	20.25	20.25	21	21.58	18.66
9	20.75	20.35	20.8	21.6	17.8
10	19.57	22.5	22.43	23.1	22.5
Mean	17.27	21.72	18.66	19.33	18.7

Body weight in gram, G3 (*L. Acidophillus*, *G4 L. casei*, *G5 L. lactis*, *Gl (positive control, L. plantarum*), G2 (control, no bacteria)

**Table 4:** Hemoglobin content (g/DL) in mice fed with *lactobacillus* sp.

Hemoglobin content before treating with bacteria. g/DL	Hemoglobin content after treating with bacteria. g/DL	Group
15.25955	14.56092	G l (positive control)
13.82552	14.19322	G2 (CONTROL)
15.03893	15.55371	G3(L. Acidophillus)
13.71521	14.74477	G4 L. casei
15.48017	16.1788	G5 L. lactis

G3 *(L. Acidophillus,* G4 L*. casei*, G5 *L. lactis*, Gl (positive control, L*. plantarum*), G2 (control, no bacteria)

**Fig. 2:**
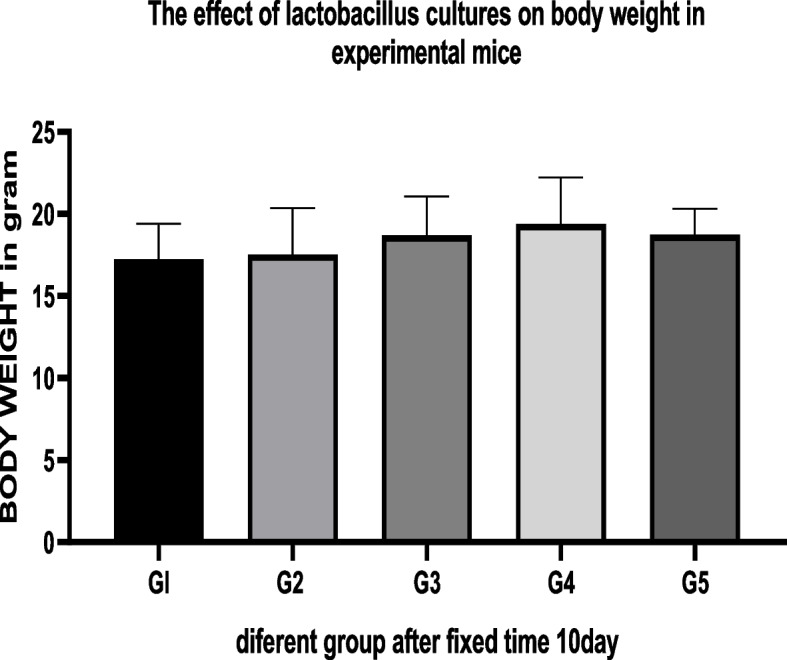
The effect of *lactobacillus* cultures on body weight in Experimental mice for 10 days

**Fig. 3:**
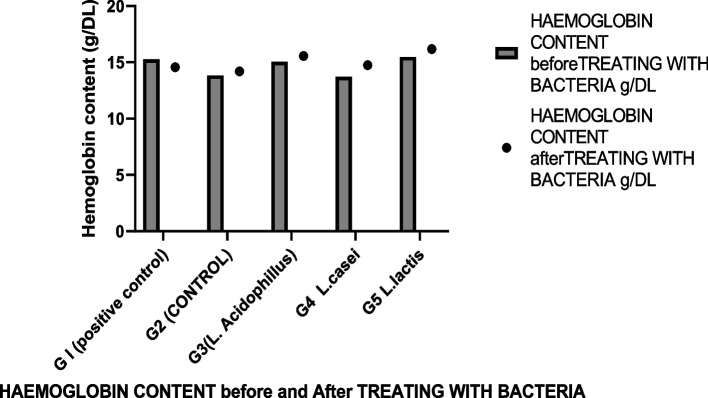
Represent hemoglobin content (g/DL) in mice fed with *Lactobacillus* sp

**Fig. 4:**
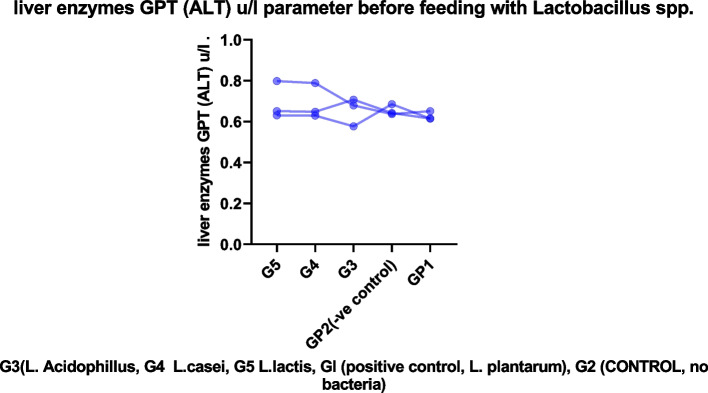
Represent liver enzymes GPT (ALT) *μ*/l parameter before feeding with *Lactobacillus* spp.

**Fig. 5:**
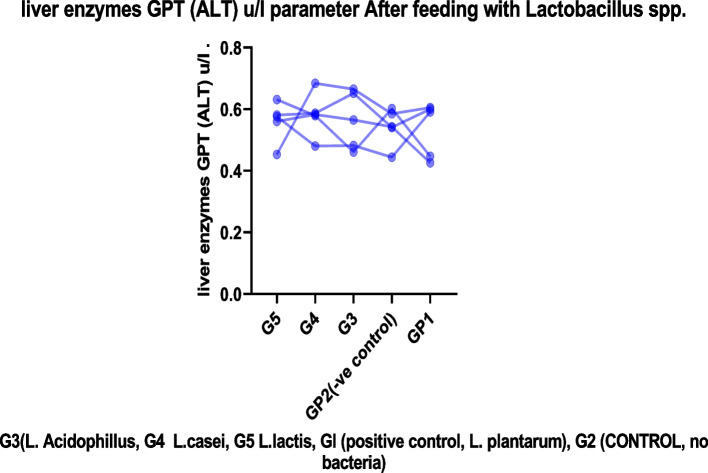
Represent liver enzymes GPT (ALT) *μ*/l parameter before after feeding with *Lactobacillus* spp.

We found no evidence of chronic toxicity under these experimental conditions based on gross pathological examination of the viscera or study of the spleen or liver weight ratios. These findings indicated that these strains, either alone or combined with their metabolites, had no obvious adverse effect on the mice's general health status, as shown in Tables 2, 3, and 4. Figures 2, 3, 4, and 5. For several centuries, LAB have been used in fermented foods and nourishes without obvious adverse effects (61). They are therefore classified as “generally recognized as safe”: GRAS [46–49].

#### Effect of *lactobacillus* spp. on liver enzyme

**Fig. 6:**
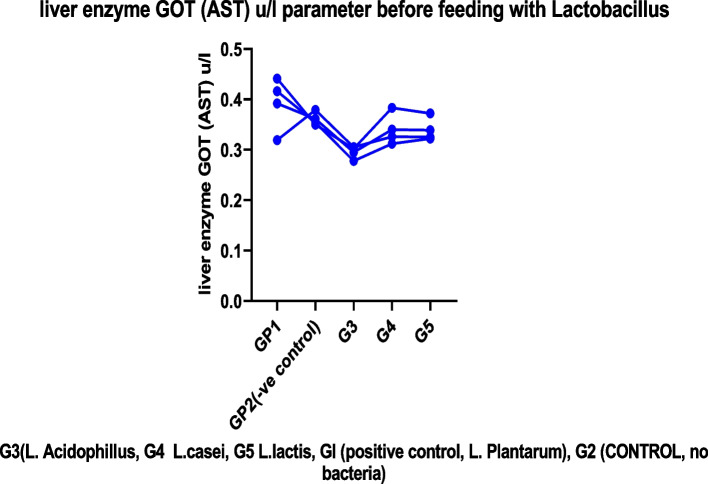
liver enzyme GOT (AST) *μ*/l parameter before feeding with *Lactobacillus*

**Fig. 7:**
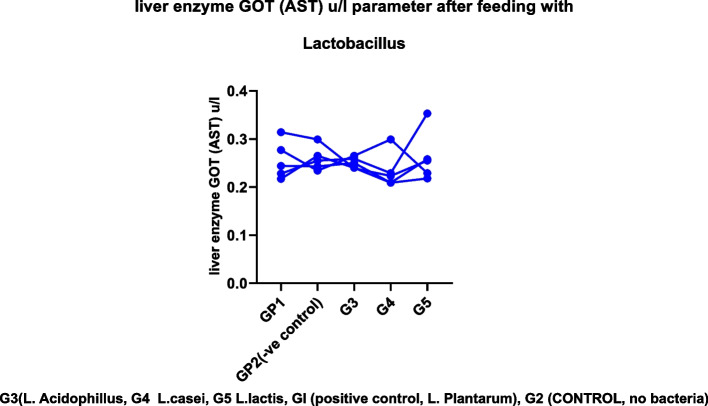
Liver enzyme GOT (AST) *μ*/l parameter after feeding with *Lactobacillus* sp

A successful growth promoter must enhance growth performance and be free of adverse side effects. For several centuries, LAB have been used in matured foods and feeds without obvious adverse effects [39, 47]. Therefore, they are classified as “generally recognized as safe”: GRAS [46, 49, 50]. Nevertheless, from the statistical study of *e* tests, we found no significant difference in liver enzyme parameters between before and after feeding as showing in Figs. 5, 6, and 7 (see Tables 5, 6, 7, and 8). When individuals have a chronic insurmountable condition, such as viral contamination, harmful injury, or alcoholic/non-alcoholic fatty liver, the serum levels of AST, ALT, and g-GTP, which serve as hepatic markers, are dramatically increased. Non-alcoholic fatty liver disease is a prevalent liver pathology encompassing a broad histologic spectrum ranging from simple steatosis to non-alcoholic steatohepatitis [12, 51]. *Lactobacillus* sp. Have been demonstrated to effectively advance liver function merely in creature show tests [39, 52]. We observed that type B yogurt contributed to a decrease in these liver biomarkers, particularly when patients with AST and ALT levels between 20 and 80 IU/L were evaluated (12–25% diminish) [53, 54].

**Table 5:** Liver enzymes GPT (ALT) u/l parameter before feeding with *Lactobacillus* spp.

Group no.	GP1 +ve control ***L.plantarum***	GP2(−ve control)	G3 ***(L. Acidophillus)***	G4 ***L. casei***	G5 ***L. lactis***
1	0.615	0.685	0.577	0.629	0.631
2	0.651	0.637	0.679	0.788	0.798
3	0.615	0.642	0.707	0.648	0.651
MEAN	0.627	0.655	0.654	0.688	0.679

G3 *(L. Acidophillus,* G4 *L. casei*, G5 *L. lactis*, Gl (positive control, L*. plantarum*), G2 (CONTROL, no bacteria)

**Table 6:** Liver enzyme GPT (ALT) U/l parameter after feeding with *Lactobacillus* spp.

Group no.	Gpl	Gp2	Gp3	Gp4	Gp5
1	0.591	0.444	0.482	0.480	0.576
2	0.605	0.585	0.665	0.684	0.453
3	0.600	0.541	0.652	0.587	0.581
4	0.447	0.602	0.461	0.579	0.631
5	0.426	0.543	0.565	0.583	0.560
MEAN	0.534	0.543	0.565	0.583	0.560

G3 *(L. Acidophillus*, G4 *L. casei*, G5 *L. lactis*, Gl (positive control, L*. Plantarum*), G2 (control, no bacteria)

**Table 7:** Liver enzyme GOT (AST) u/l parameter before feeding with *Lactobacillus*

No.	Gpl	Gp2	Gp3	Gp4	Gp5
1	0.416	0.354	0.278	0.312	0.322
2	0.441	0.350	0.302	0.383	0.372
3	0.319	0.379	0.305	0.326	0.325
MEAN	0.392	0.361	0.295	0.340	0.339

G3 (*L. Acidophillus*, G4 *L. casei*, G5 *L. lactis*, Gl (positive control, L*. Plantarum*), G2 (control, no bacteria)

**Table 8:** Liver enzyme GOT (AST) u/l parameter after feeding with *Lactobacillus*

No.	GP1	GP2	GP3	GP4	GP5
1	0.244	0.243	0.250	0.209	0.258
2	0.217	0.265	0.240	0.209	0.218
3	0.314	0.299	0.239	0.223	0.255
4	0.277	0.234	0.265	0.299	0.229
5	0.228	0.255	0.259	0.229	0.353
Mean	0.256	0.259	0.251	0.234	0.259

G3 (*L. Acidophillus*, G4 *L. casei*, G5 *L. lactis*, Gl (positive control, L*. Plantarum*), G2 (control, no bacteria)

Sort A yogurt decreased the ALT value. The current study is the primary report of a trial in which a certain *lactobacillus* strain was found to move forward liver function. Another study revealed that Probiotic isolate possesses the highest potential of (48%) cholesterol reduction compared to the other isolates. Thus, the use of these LAB isolates for yoghurt-making can offer the value addition of lowering cholesterol and vitamin B12 fortification in fermented food [23, 55–57].

## Conclusion

The present study showed that the dairy product is a source of potential probiotic strains of LAB. The isolates meet several functional features to be considered a suitable probiotic for application in food fermentation where isolated bacteria can tolerate acidic medium bile salt, a favorable enzymatic activity, and no hemolytic activity. So, we consider it a great potential probiotic character and safe for human use. There is prove that probiotic strains utilized as commercial microorganism are safe for utilize and considered a successful growth promoter enhances growth performance and is free of adverse side-effects. The security of probiotic items is evaluated based on the phenotypic and genotypic characteristics as well as measurements of the microbe characterize. *Lactobacillus* besides the gene responsible for hydrolyzing bile salts confirm the probiotic character of superior isolates and suggesting two hypotheses trying to explain the mechanism. One of them is that bacteria may bind or incorporate cholesterol directly into the cell membrane the other one is, bile salt hydrolysis enzymes deconjugate the bile salts, which are more likely to be exerted, resulting in increased cholesterol breakdown.

## Data Availability

Please contact author for data requests.

## Abbreviations

LAB: Lactic acid bacteria
CFU: Count forming unit
GRAS: Generally recognized as safe
IU: International unit
nmol: Nano mole
